# From Blackboard to Green Screen: Delivering a Semester of In-Depth Database Lectures via *YouTube*

**DOI:** 10.1007/s13222-020-00362-8

**Published:** 2020-12-29

**Authors:** Torsten Grust

**Affiliations:** Department of Computer Science, Database Systems Research Group, University of Tübingen, Tübingen, Germany

**Keywords:** Database course, YouTube, Live coding, SQL

## Abstract

We report on the conversion of two advanced database courses from their classical in-lecture-hall setup into an all-digital remote format that was delivered via *YouTube*. While the course contents were not turned on their heads, throughout the semester we adopted a video style that has been popularized by the *live coding* community. This new focus on the live interaction with the underlying database systems, led us (1) to adopt the idea of SQL probe queries that are specifically crafted to reveal database internals and (2) a study of database-supported computation that treats SQL like a true programming language. We are happy to share videos, slides, and code with anyone who is interested.

## From Blackboard to Green Screen

To help keep the *COVID-19* pandemic at bay, during the summer semester of 2020 all lectures at the University of Tübingen were held in an online-only format. This also applied to the two courses *“Implementation of Database Systems”* (referred to as $\textit{DB2}$ in the following) and *“Advanced SQL”* which I read during that term. Foregoing the established in-lecture-hall teaching and going fully digital affected all aspects of these courses. Throwing the existing course material overboard simply was no option due to limited preparation time. Instead, I opted to strike a middle ground based on existing slide sets and code examples: delivering the material in terms of *YouTube* videos required a shift of focus and reshuffling but did *not* turn the courses on their heads. The end result were two variants of $\textit{DB2}$ and *Advanced SQL* which I consider the best I have yet delivered–depth was gained (not lost) and an extensive archive of video and code material was created that I am happy to share with everyone.

The present article shines a light on what it meant to read the courses in front of a green screen instead of a blackboard (Sect. 2) and zooms in on details that made $\textit{DB2}$ (Sect. 3) as well as *Advanced SQL* (Sect. 4) work on *YouTube*.

## Lecturing and Live Coding on *YouTube*

**Fig. 1:**
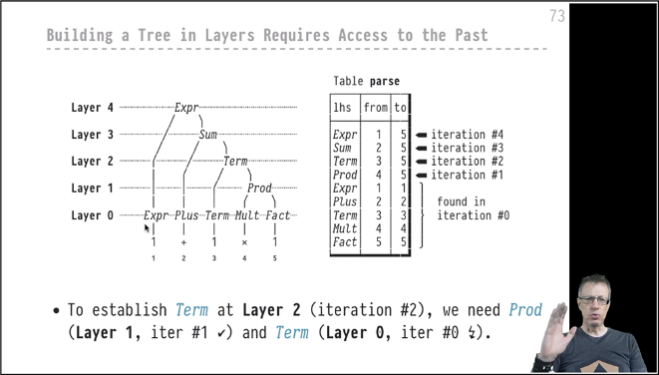
Slide-only scene with talking head. The (parse) tree and table on the slide are exclusively typeset using *PragmataPro* characters

The backbones of both courses have always been two extensive slide decks, deliberately designed to be heavy on illustrations and light on text. Since around 2016, I author all slide decks via (1) *Markdeep* [13], a Markdown variant that renders quite intricate drawings directly from textual descriptions that are placed *inline* with all other slide text, and use (2) *PragmataPro*, a font whose 9000+ characters support to lay out code, tables, trees, graphs, and other regular structures (see Fig. 1). *Markdeep*-based slides render instantly in any Web browser. The purely text-based input facilitates slide versioning and collaborative authoring (*e.g.*, via *git*), encourages hot fixes, and allows to keep slide text as well as associated code samples together. We will see that the latter was crucial. These slide decks were largely kept unchanged, yet bugs were fixed and adaptations to the then current versions of the database systems (*PostgreSQL* v12 and *MonetDB* 5) in use were applied.

Resting on the foundation of these slide sets provided a good start. Still, an online course entirely based on these decks, essentially consisting of hour after hour of narrated slides in a series of *YouTube* videos, was a dire prospect for both, students and myself. A closer look at the core contents of both courses turned things around:

- $\textit{DB2}$ explores the internals of database systems through the submission of *probe (SQL) queries* and subsequent study of which system components interacted exactly how to respond to these probes.
- *Advanced SQL* treats SQL like a *true programming language* that can be used to express (very) complex computation close to the data.

Both courses naturally lend themselves to **a lecture format that uses code extensively** (predominantly SQL, but also C, the systems’ implementation language) to develop and express the central ideas, formulate examples, and devise experiments. A focus on code and the delivery via *YouTube* led to a course redesign that revolved around **live coding**. There is a vibrant *YouTube* subculture on live coding in many forms, and I entirely banked on its success from the first to the last lecture video. The approach held the promise that it would be far more engaging than the slide-only lectures. The evaluation of both courses by my students–most of which were a experienced live coding audience, it turned out–showed that that promise was kept (see Sect. 5). *Truly live* coding also kept me on my toes during the semester, in the best possible sense.

**Fig. 2:**
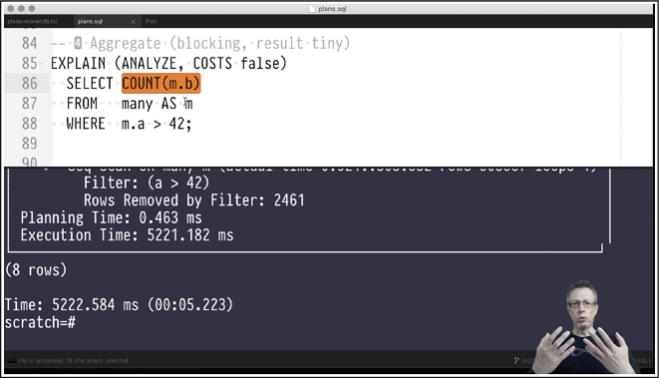
Scene with a split-editor view (SQL code in the top half, the *PostgreSQL* REPL at the bottom). Code was edited and run live

**Canned video, live coding.** Given the nature of the courses, live coding happened in the interactive *read-eval-print-loops* (or REPLs) psql and mclient of *PostgreSQL* and *MonetDB*, respectively. Less often, the unix shell was used to compile and run C code or initiate benchmarking runs. To save time and not bore viewers with the authoring and mistyping of larger code fragments, I used a text editor with a split view: code was rearranged and completed in the split’s top half, marked for evaluation, and (on key press) submitted to the bottom half which hosted the current REPL (see Fig. 2 showing a SQL query on the top and the [tail of] its EXPLAIN output in the bottom psql shell). In the *Markdeep* source, these code fragments live right next to the slide text they relate to. This helps to keep both in sync and provides welcome context for a piece of code that may have been written months or years ago. Before shooting the video, I cut&pasted the relevant fragments out of the *Markdeep* source into a blank file such that (1) viewers could focus on the code alone and (2) a code-only file could be distributed to the students: everyone was able to replay the code experiments on their own machines without the need to copy verbatim code from slides (or, let alone, *YouTube* streams). Call-outs in the videos explicitly named the current code file in use, making it immediate for viewers exactly which piece of the distributed material currently was in focus.

During the video shoot itself, I constantly switched from slides to code and back. It was absolutely instrumental that this switch was instant and seamless. Video recording and streaming software supports such multi-scenario setups; we come back to these at the end of this section. To tie slide contents and code samples tighter together, I used the *PragmataPro* font to typeset and edit both, leading to identifiers and symbols on the slides that were instantly recognizable in the live code. *PragmataPro* is highly legible yet narrow which ensured that code fragments remained readable even if the *YouTube* streams were watched at lower resolutions. At the same time, this allowed slides as well as editor windows to show reasonably-sized portions of code despite my deliberate use of large fonts throughout.

While videos were recorded in advance and then uploaded to *YouTube* (see below), the coding during the shot itself was truly live: I edited and ran code, applying modifications and extensions to it when they crossed my mind during the shoot. Such *ad-hoc* code changes led to some of the most insightful experiments during the semester but, inevitably, also conjured syntactic or logic errors here and there. I fixed these during the shoots while commenting on my interpretation of error messages. Quite quickly student feedback made it clear that this was seen as insightful rather than a nuisance. In consequence, I relied on pre-recorded coding footage only once during the semester, when an experiment based on CPU caching and branch prediction behavior turned out to be so time-sensitive, that the simultaneously running video recording process would ruin all measurements.

As intimidating and daunting the live coding sections felt at first, the more I enjoyed them towards the end of the semester. Knowing that true boo-boos could be removed through post-shoot video editing helped but was seldomly necessary. The frequent switches from slide to code and back led to changes of pace, structured the video’s narrative, and allowed for interactive bits of content that slides could never contain. Sects. 3 and 4, respectively, discuss how the $\textit{DB2}$ and *Advanced SQL* courses were laid out to emphasize the live coding parts.

**Push and pull: weekly assignments.** The prevalence of code and the time spent to develop, modify, and discuss fragments of SQL and C during the videos, also set the tone for the weekly assignments that accompanied the entire semester: students outright expected to read and write significant chunks of code during these assignments. With essential support by my assistants, we made sure that the coding style (*e.g.*, code formatting and choice of identifiers) in the assignments matched those in the lecture videos. After a while we found that many students submitted code that mimicked the lectures’ style, leading to well-structured submissions that were easy to grade by assistants and tutors.

For the $\textit{DB2}$ course, the availability of the source code for both, *PostgreSQL* and *MonetDB*, facilitated new kinds of assignments. Once a lecture had identified an algorithm or data structure as a central database kernel concept, we asked the students to locate its implementation in the systems’ source. Invariably, this turned up variations or simplifications of the original textbook concept, which helped to appreciate the complexity of a true and tested DBMS.

Just like the lecture slides and associated code, we published the assignments on *GitHub*. Students pulled the assignment text along with code skeletons and completed the latter to build their solutions in teams of two, using *GitHub*’s collaboration features (*e.g.*, pull requests). Tutors then annotated the solutions, pushing the graded submissions back to *GitHub* from which the students pulled and reviewed the tutors’ comments. The tutors’ ability to place annotations *right next to or inline with* the students’ code led to very specific and actionable feedback, sometimes in the form of actual code snippets that would improve or correct the students’ original solution. *GitHub*’s push-pull model turned out to be a very fitting two-way communication channel that helped to establish a connection between students and their tutors, making up at least partly for the lack of face-to-face meetings.

Throughout the entire semester and beyond, we operated a *Discourse*-based forum that served as a hub for discussion of the video lectures and the weekly assignments. Any new posting in that forum would trigger a notification such that my research assistants and myself were able to respond immediately, typically within less than 30 minutes. This led to a lively atmosphere of back-and-forth in the forum with several hundreds of postings during the overall semester. While even a bustling forum is hardly a replacement for live interaction, polls among the students showed that, taken together, (1) a forum with quick turnaround, (2) the around-the-clock availability of *YouTube*, and (3) the ability to pause/rewind lecture videos as needed, formed an acceptable replacement for in-class tutorial sessions.

**Think like a *YouTuber*.** Staying alert and attentive throughout a 90-minute in-hall lecture is challenging. To expect the same from viewers of a video stream at home–where distraction is abundant–is straight out wishful thinking. From the get go, I thus aimed for brief lecture videos that averaged about 25 to 30 minutes in length (looking back, I now regard this as the absolute maximum). While this payed respect to limited attention spans and helped to blend with the “*YouTube* *culture*,” there also were ample practical benefits:

- The resulting video recordings were of manageable file size, typically less than $1\,\text{GB}$. This simplified file management operations and backup.
- Video editing software faced manageable file sizes and remained snappy. In effect, video post-production time shrank.
- Video upload to *YouTube* was a matter of seconds. *YouTube*’s own encoding process for HD video completed in mere minutes.

Further, limited video length enforced a bite-sized presentation of the course contents: it helped to entirely focus on the $\text{B}^{+}$tree leaf level now, take a breath, and zoom into inner nodes only later instead of tackling a monolithic all-embracing $\text{B}^{+}$tree discussion. Student feedback was unanimously positive.

For $\textit{DB2}$, the entire course contents was split up into 83 videos; for *Advanced SQL*, 58 videos were shot. *YouTube*’s *play lists* turned out to be ideal to organize these collections: viewers let their video players walk the list from front to back to take in the entire contents. In addition, I was disciplined in numbering all videos, gave descriptive titles, and added keywords which facilitated to hop and search through the play lists at will. Weekly assignments then referred to these video numbers (*“Relevant videos for this assignment: #*$n$* to #*$m$*”*). Students used these hints to adjust the rate at which they consumed the play lists.

Not least important, a video length of about half an hour helped me to keep my concentration throughout the shoot. The intricacies of the upcoming slides (way less than 10 in one video, typically) and code fragments to be discussed in that time frame, were easily remembered. As a consequence, almost all videos were shot in one take from start to finish, a process that would not last much longer than the 30-minute target. Still, close attention and care was required. After about four to five video shoots, I started to feel exhausted and adjusted my daily pensum accordingly.

**Fig. 3:**
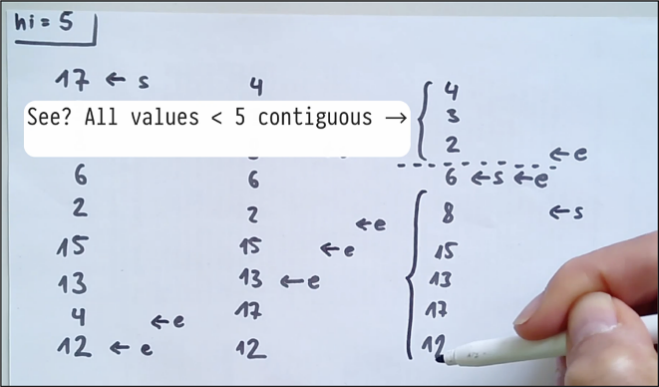
An overhead camera captures quick scribbles on a blank sheet. The temporary call-out was added during video post-processing

Video post-processing was kept to be very light and typically required less than 15 minutes per video. Occassionally, one of the above-mentioned boo-boos needed cutting–the ability to insert fluid frame-to-frame transitions made most of these cuts next to invisible. Whenever that appeared helpful, I overlayed *call-outs* onto a sequence of frames (see Fig. 3). These call-outs pointed out details I forgot to mention during the shoot, contained corrections, or directed viewer attention to a particular portion of the frame. Used sparingly, these can be effective tools to touch up an already canned recording. Call-outs also worked great to sprinkle the videos with the occasional joking remark.

The video recording itself was performed with *OBS*^1^, a piece of free (in the beer as well as the GNU sense) software that I cannot recommend highly enough. To set the stage, I defined multiple screen layouts (scenes) between which *OBS* could switch on a key press: intro as well as outro screens, slides, and editor + REPL. I additionally set up a scene for an overhead camera that would film my hand while I was scribbling on a blank DIN A4 sheet (Fig. 3). Now and then I used this additional camera to capture quick sketches or examples that would benefit from a stepwise development in front of the viewers.

Most of the scenes were configured to insert a camera picture of my head and upper waist into the frame’s lower-right corner as shown in Figures 1 and 2. Since such an insertion unavoidably obscures parts of the slide or REPL contents, I tried to minimize its impact and removed the background behind me using a vanilla green screen. I am fully aware that the value of *talking heads* is up for debate. Over the course of the semester, I found myself to make more and more use of the feature, though: it was essential that my arms and hands were captured which allowed me to pinpoint caveats (), rate a specific behavior of the database system (/), or use similar gestures. The talking head definitely helped to keep *myself* engaged and student feedback suggests a similar effect on the viewers’ side.

Let me not close this section without stressing the importance of good lighthing and audio recording. Two LED light panels with adjustable white/yellow tint allowed me to continue shooting even if exterior light was dim, *e.g.*, due to weather or time of day. The face and overhead cameras definitely benefited from consistent and homegenous illumination. However, the key piece of the equipment puzzle has been a professional microphone. Installed on an extendible arm, I adjusted the microphone to be next to my mouth, just outside the camera frame. Regarding audio quality, the microphones built into off-the-shelf computers are simply no match: in comparison to the studio mic, you sound as if you were sitting in a tin can, honestly. If you can invest into a single piece of recording equipment only, make it the microphone.

## *DB2*: Exploring Database Systems Internals Through Query Probes

( Play list: http://tiny.cc/DB2-summer-2020.)

As its name already suggests, $\textit{DB2}$ is the second installment in a tandem of courses on relational database technology. $\textit{DB1}$ introduces students to a variety of data models with the relational model being front and center. These data models are never discussed without their associated languages. Indeed, the lion share of $\textit{DB1}$ revolves around SQL. While the language itself is a subject of core interest, SQL is also used to explore other fundamental concepts of the relational model–functional dependencies, say–in terms of queries.

$\textit{DB2}$, then, is meant to look behind the scenes and explore the internals of systems that *implement* the relational data model. With an audience in mind that has heard $\textit{DB1}$, the design of $\textit{DB2}$ builds on a working knowledge of SQL: **the SQL language is used to formulate a series of queries and updates that are crafted to exercise the individual components of a database system.** The system’s built-in or tacked-on monitoring facilities are then used to observe the database kernel’s response. This naturally leads to a study of the kernel’s algorithms and data structures involved in generating the result of our queries.

This series of SQL *probe queries* replaces the commonplace walks through architectural “boxes-and-arrows” diagrams that determine the structure of most $\textit{DB2}$-like courses. Instead, we bank on the natural curiosity felt by novice and not-so-novice users of SQL: *“How did that query execute in only 5 milliseconds?”*, or *“Table size only doubled but the query now used significantly more buffer space–why?”* If the series of probe queries is arranged carefully, students will encounter database components in a gradual fashion. Importantly, the presence and function of the components is motivated by the probe query itself. Juggling with probe queries further provides essential training for database practice beyond the course: posing the right inquisitive queries can disclose lots about any RDBMS’s internals. The overarching rationale here is that the immediate application of existing knowledge (SQL) is more engaging and satisfying than the confrontation with the overwhelmingly complex architecture of database components, the operation of which is (initially, at least) opaque.

The $\textit{DB2}$ material has been derived from a variety of scientific papers, few textbook excerpts, developer blogs and mailing list postings, *Stack Exchange Q&As*, SQL references and standards, RDBMS documentation and kernel source code, as well as experience and best practices. A dedicated chapter on the choice of indexes that fit a given set of queries was inspired by Markus Winand’s book *SQL Performance Explained* [20]. This book is remarkably to-the-point, uses clever notation to illustrate the value distribution inside index pages, and is ripe with practical advice on index definition and usage in contemporary SQL. A free edition is available on the Web [21].

### SQL Probe Queries

A course design based on the probe query idea necessarily emphasizes (1) the formulation of numerous, typically brief, SQL snippets and (2) the collection and analysis of the system’s monitoring output. Both made $\textit{DB2}$ a great match for the live coding style of the *YouTube*-based lecture format.

In the edition of Summer 2020, $\textit{DB2}$ put the spotlight on *PostgreSQL* and *MonetDB*, two RDBMSs that implement the relational data model, but occupy almost diametrically opposed points of the design spectrum. *PostgreSQL* [14] is a classical representative of page-based row storage for wide tables over which a Volcano-style query engine evaluates a rich dialect of SQL. *MonetDB* [3] holds binary tables (BATs, column vectors) in main memory and relies on the operating system to page memory in and out as required. Query evaluation materializes all intermediate results and is tailored to suit modern multi-level cache and CPU architecture.

If possible at all, we submitted the *same* query probes to both *PostgreSQL* and *MonetDB*. This nicely highlighted the consequences of the systems’ fundamentally different design decisions. I am convinced that the probe query paradigm is particularly good fit for a course that explores multiple RDBMSs in parallel.

**Table 1:** Excerpt of probe queries used in the $\textit{DB2}$ course. Each $Q_{i}$ represents a family of concrete, executable SQL queries

**Q**	**SQL probe query** (excerpt)	**Components triggered/Concepts explained**	
		***PostgreSQL***	***MonetDB***
$Q_{1}$	SELECT u.* FROM unary AS u	Heap files and sequential scan, block I/O on secondary storage, HDD/SSD access time, free space management	Simple MAL programs, memory mapping (mmap()), positional access into vectors
$Q_{2}$	SELECT t.* FROM ternary AS t	Row storage, heap file page layout	Full vertical table fragmentation, positional BAT joins
$Q_{3}$	SELECT t.a,t.c FROM ternary AS t	Row layout and field access, padding and alignment, NULL (non-)storage	Column vector (non-)access, cache pollution
$Q_{4}$	INSERT INTO/UPDATE/DELETE FROM …	Plan operators Seq Scan, Update, …, row versions (MVCC), row visibility and timestamps, VACUUM	Delta tables, visibility of changes, delayed changed propagation
$Q_{5}$	$\langle\textit{sequences of queries, e.g.,}Q_{3};Q_{1};Q_{3}\rangle$	Temporal and spatial locality, buffer cache, page replacement	CPU cache hierarchy, predictable memory access, prefetching
$Q_{6}$	SELECT $\langle\textit{complex expression}\rangle$	Expression representation and interpretation, JIT compilation	Sequential evaluation *vs.* data flow, tight loops, loop unrolling, SIMD parallelism
	FROM ternary AS t		
$Q_{7}$	SELECT … FROM ternary AS t	Predicate evaluation, selectivity, predicate simplification	Selection vectors, control flow, branch (mis-)prediction, branch-less selection
	WHERE $\langle\textit{predicate}\rangle$		
$Q_{8}$	SELECT … FROM ternary AS t	Index support, Index Scan, ordered indexes (B${}^{+}$tree), inner/leaf nodes, clustered indexes, Bitmap Scan, $\text{B}^{+}$tree maintenance	BAT properties (ordering), tactical optimization, order indexes, cracker indexes
	WHERE $\langle\textit{indexed predicates}\rangle$		
$Q_{9}$	SELECT … FROM ternary AS t	Indexes on expressions, composite indexes, matching queries and indexes, partitioned $\text{B}^{+}$trees, string pattern matching, partial indexes, index-only query evaluation, sorting with $\text{B}^{+}$trees	–
	WHERE $\langle\textit{complex predicates}\rangle$		
$Q_{10}$	SELECT … FROM ternary AS t	Blocking plan operators, external merge sort, replacement sort, sorting *vs.* hashing, parallel grouping/aggregation	Order indexes, tactical optimization, TIM sort, sort refinement
	GROUP BY …/ORDER BY …		
$Q_{11}$	SELECT … FROM one AS o, many AS m	Join algorithms (nested loops, indexed nested loops, merge, hash, hybrid hash)	Join indexes, BAT partitioning, radix cluster join
	WHERE $\langle\textit{join predicate}\rangle$		
$Q_{12}$	$\langle\textit{complex multi-clause SQL{} query}\rangle$	Operator orchestration, Volcano-style on-demand evaluation, pipelined operators, SQL cursors	Full materialization, MAL instruction scheduling, data dependencies and parallelism
$Q_{13}$	$\langle\textit{complex TPC-H join query}\rangle$	Reading complex EXPLAIN plans, query normalization, query unnesting, join tree optimization, cost model, cost of plan operators	–

**A selection of SQL probe queries.** Table 1 reviews an excerpt of the probe queries and the RDBMS components that were triggered upon execution. In this overview, MAL refers to the *MonetDB* *Assembly Language*, the system’s internal query representation. Identifiers unary and ternary refer to single- and three-column tables, respectively. Likewise, one and many denote a pair of tables whose rows are in a *one-to-many* relationship.

The course defines an entire family of such “playground tables” whose deliberately simple and column and row sets are tuned to support particular probe queries. A similar remark appplies to the probe queries themselves. You will find that query complexity grows as you walk down Table 1: the queries aim to use the minimal number of constructs and complexity required that already triggers the RDBMS component in focus. The simpler the queries, the easier can the system’s response be interpreted.

The probe query set is subject to extension, obviously. In Summer 2020, regrettably, there was little time to address transaction management. An upcoming $\textit{DB2}$ edition will likely employ pairs of probe updates that refer to the same database objects to demonstrate the effects of isolation levels, locks, or recovery logs.

The focus on code in $\textit{DB2}$ suggested a hands-on style even if SQL was of no immediate help in exploring specific system internals and phenomena. Examples include:

- The manual construction of *MonetDB* MAL programs, providing a sense of how close query compilation and compiler construction, code generation in particular, really are.
- The development of C code that mimics *MonetDB*’s tight core loops, an ideal scenario to experiment with the effects of branch prediction or memory prefetching in modern CPU and memory architectures.
- The browsing of *PostgreSQL* and *MonetDB* C source code fragments. The quality of the former, in particular, is remarkable: well-documented, consistently layed out, with several algorithms implemented in their original, almost textbook-style, form. *PostgreSQL* source would make for a worthy subject of study in any software engineering course.

### “X-Ray Imaging” of RDBMSs

Database systems are not as black a box as they are often perceived. Most come equipped with a number of monitoring and logging facilites off the shelf. *PostgreSQL*, in particular, is to be applauded for its countless extensions that open up additional sideways entries and provide new views of its internals. It is commonplace for these extensions to offer an API that is directly accessible from *ad-hoc* SQL queries. Here, too, students are able to put acquired SQL skills to good use. I regard *PostgreSQL* as *the* ideal vehicle for a $\textit{DB2}$-like course for this reason. (*PostgreSQL* also is a perfect host for *Advanced SQL* as we will elaborate on in Sect. 4.)

To make this point, the $\textit{DB2}$ edition captured on *YouTube* used the following means to study *PostgreSQL*’s response to the probe queries:

#### Extension pageinspect:

Renders detailed page layout in heap (data) and index files. Provides all means required to re-enact $\text{B}^{+}$tree root-to-leaf traversal. Exposes internal organization of individual rows, including *NULL* bitmaps and storage of variable-width columns.

#### Extension pg_freespacemap:

Displays available space on heap file pages. Explains allocation of rows on *INSERT*.

#### Extension pg_visibility:

Makes row visibility map of heap files accessible. Contains vital information used to guide optimizer decisions for/against index-only scans.

#### Extension pg_buffercache:

Provides tabular view of buffer cache contents, along with details that guide the system’s page replacement strategies (dirty bits, page usage counters). Makes behavior of LRU and ring buffering tangible.

#### Extension pg_stat_statements:

Tracks execution statistics for *all* SQL statements executed. Includes blocks read/written/dirtied, rows touched, invocation counts, detailed timings, *etc.*

#### Extension pgstattuple:

Page-level statistics of row size, fraction of invisible rows, and free space.

#### SQL’s EXPLAIN facility:

Arguably the central tool. $\textit{DB2}$ invested extra time to make sure that students learned to read the finer details of query plans, including repeated execution (loop) or non-execution of sub-plans, estimated row size (width), buffer allocation, buffer hits and misses, divergence of response and evaluation time, and JIT code dumps (LLVM bitcode).

#### Planner control (enable_*):

Disable specific plan operators to influence optimizer decisions. *e.g.*, enforce/forbid Index Scan to illustrate its impact on plan performance, affect query unnesting, prescribe join order. To take full control over plan generation, we have developed *PgCuckoo* [9] which admits the manual construction and injection of sub-plans into *PostgreSQL*. (This was not used during Summer 2020.)

#### Cost model control:

Turn tuning knobs of the cost model (like seq_page_cost or cpu_tuple_cost) to study its impact on plan generation and simulate specific system configurations. *e.g.*, in-memory operation.

#### Hidden table columns:

Expose system-maintained row details for inspection in regular SQL queries, including row IDs (ctid) or MVCC row validity timeframes (xmin, xmax).

#### Config file postgresql.conf:

*PostgreSQL*’s sizable configuration file affects all core system aspects, from buffer size to fine-grained control over plan parallelism.

The list is shorter on the *MonetDB* side, but the system still provides essential looks behind the curtains:

#### SQL’s EXPLAIN facility:

*MonetDB* exposes the MAL programs that implement SQL queries. MAL code may be authored and submitted for execution via the mclient REPL.

#### SQL’s PLAN facility:

As an intermediate form between SQL and MAL, the system generates rather conventional algebraic plans which PLAN renders in the REPL.

#### mserver5 runtime options:

The *MonetDB* server process can be instructed to verbosely log details on a variety of runtime aspects. Option --algorithms makes tactical optimization decisions explicit, for example.

#### The stethoscope:

Attaches to the server process to provide fine-grained MAL profiling information and instruction traces. (Not used during Summer 2020.)

**Fig. 4:**
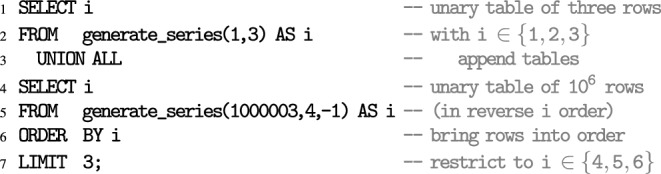
Listing – SQL query that reveals the presence

Beyond these facilities, a little creativity can go a long way to lay bare RDBMS internals and make them tangible at the SQL prompt. The timing of successive SQL cursor operations, for example, lets students experience the effects of Volcano-style on-demand pipelining [8] or blocking plan operators. To illustrate, timing the first five FETCH NEXTs on a cursor defined over the SQL query of Fig. 4, will reveal ORDER BY (and thus plan operator Sort) to be blocking.

As a last resort, both *PostgreSQL* and *MonetDB* were subject to code instrumentation and subsequent recompilation. We used this to inject logging instructions that revealed the sets of access paths maintained by the *PostgreSQL*’s optimizer during join plan generation.

At the end of the semester, we found that the extensive use of these monitoring hooks made the students significantly more confident in dealing with the formerly black boxes. Some “RDBMS magic” was lost underway, but this is a price we were happy to pay.

## Advanced SQL: Teaching SQL Like a True Programming Language

( Play list: http://tiny.cc/AdvSQL-summer-2020.)

“Can you express $\langle\textit{computational problem }P\rangle$ in SQL?” For arbitrary $P$ and for more than 20 years now, since the advent of SQL:1999 [16], the answer to this question is a definite “yes”: the introduction of recursive common table expressions (CTEs) has turned SQL into a Turing-complete language. The question remains whether $P$ indeed *should* be tackled using SQL. The knee-jerk answer goes along the following lines:

1. Use SQL if $P$ is “query-like,” *i.e.*, if the iteration/filtering/aggregation of data collections is prevalent in the computation.
2. Otherwise, rather use a real programming language.

The course *Advanced SQL* has been designed to challenge this far too common point of view. We set out to use SQL in earnest and consider a substantially larger subset of problems $P$ to be in its reach. *Advanced SQL* does *not* advocate to replace general purpose programming languages, but **treats SQL as a true programming language with very specific strengths** (and, not the least, terseness and style). The course is ripe with scenarios and use cases that would not be considered “query-like” by most, but still have elegant and efficient SQL solutions. A variety of these problem scenarios is listed below. We pursue the goal to boost the students’ ability to think and write in SQL, certainly way beyond the SELECT-*FROM*-*WHERE* class of problems.

A division of complex computation between the database system (where the data lives) and an external programming language (where the processing takes place) is bound to suffer from the infamous DB/PL bottleneck [5]. In the face of ever more workloads that are data-intensive *and* computationally complex–think database-supported machine learning [2]–I do think that *Advanced SQL* is a timely course offering: *move your computation close to the data* [15] has never been a more important credo.

Straight in line with the overarching theme of this article, *Advanced SQL* promotes to read and write lots of SQL code. Since some of the computational problems we tackle are algorithmically complex, the associated SQL text may indeed be substantial in size. At points, *Advanced SQL* devotes entire series of slides to develop a single query. The course thus discusses techniques to author large queries, predominantly based on the use of common table expressions (WITH) in which, helpfully, the reading and evaluation order of SQL aligns. Much like for $\textit{DB2}$, this code-centric approach in the lectures also determines the style for the accompanying assignments: students author many-line SQL statements and the lectures slides and videos offer methods to organize the resulting code.

The focus of *Advanced SQL* deliberately is on the syntax, semantics, pragmatics, and–first of all–practical use of SQL. Aspects of the language’s implementation or optimization are not on the table: this is the realm of $\textit{DB2}$. We aim for efficient formulations but, for once, this takes the back seat behind expressiveness, readability, and good style. By the end of the course, some may argue that we have crossed the borders of what should be expressed in SQL–this may apply to the use of non-linear recursion which is perfectly expressible and usable in *PostgreSQL*, for example. Here, our rationale is to bend the language until it breaks: knowing SQL’s limits helps to assess its applicability.

*Advanced SQL* uses *PostgreSQL*’s dialect of SQL. The system offers a stable, well-documented, and complete implementation of SQL:2003 [17] that is constantly being modernized. In particular, recursive CTEs in *PostgreSQL* come with less restrictions than their counterparts in *Oracle* or *Microsoft SQL Server*. I used *PostgreSQL* v12.1 in the videos of the Summer 2020 course, but any version 11 or beyond would have been workable.

**Fig. 5:**
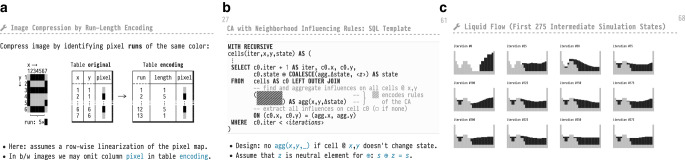
Excerpt of the slide material (focus on window functions and recursion) developed for the *Advanced SQL* course. **a** Run-length encoding of a pixel image. **b** Piecemeal construction of a complex query. **c** States of a SQL-based liquid simulation

**SQL beyond **SELECT**-**FROM**-**WHERE**.** True to its denomination, the course only spends the first few lectures to remind students of the basics of the relational model and the SELECT-FROM-WHERE backbone of SQL. The course then dives deep into advanced ideas in SQL, among these:

- SQL binds **row variables** to row values of row types. More core rather than an advanced fact, remembering it is key to appreciate concepts like subquery correlation or LATERAL bindings.
- Table **cells may hold values of complex type** $\tau$ (*e.g.*, arrays or JSON objects) and SQL is equipped with functions that convert between such cell contents and tables of simple values. This suggests a general methodology to turn a SQL into a processor for $\tau$-typed data:
  1. Unfold $\tau$ values into regular tables. If $\tau\equiv\text{array}$, then this is readily performed via table-valued function *unnest()* jointly with WITH ORDINALITY.
  2. Use SQL constructs to process these tables.
  3. Fold the resulting table back into $\tau$ values, carefully re-establishing the complex stucture of $\tau$. For $\tau\equiv\text{array}$, use an ordered array_agg() aggregate.
  This multi-staged approach naturally leads to the use common table expressions early on in the course.
- For any row, **window functions** provide fine-grained access to its immediate or even remote *row neighborhood*. This admits the elegant expression of a whole family of algorithms based on *scans* or *parallel-prefix computations* [1].
- Common table expressions can express **iterative or recursive computation** [6]. SQL’s fixpoint-based semantics is a perfect fit for many algorithms–if it is not, bookkeeping can be introduced systematically to maintain algorithm state and ensure termination. This discussion constitutes the course’s core part.
- **PLSQL** interleaves set-oriented SQL evaluation with the statement-by-statement execution of side-effecting commands. Most developers are well-versed in this imperative style of programming. However, it comes with the substantial cost of a constant back and forth between the SQL and PL/SQL execution modes [4]. PL/SQL thus cannot provide a general one-stop answer to database-supported computation.

**Complex computation close to the data.** Coverage of the above SQL concepts, however, is not what primarily determines the flavor of *Advanced SQL*. Rather, the course focuses on algorithmic challenges and then identifies the SQL constructs required for their solution. To frame these challenges, extra effort was invested to devise scenarios off the beaten paths–not the least, this facilitated the production of slides and varied assignments. I made a point to *never* use scenarios like the well-trodden employees-departments-projects: these are overused, uninspired, and hardly suited to engage students in deep thinking and query authoring.

A selection of the algorithmic problems discussed in *Advanced SQL* is found below [in brackets, we point at SQL constructs that helped tackle these problems]:

### Shape scanner:

Given an unknown shape in the two-dimensional plane, perform a scan to trace its border. Use these traces to render the shape. [Non-recursive CTEs, iteration via table-valued function generate_series(), geometric objects and operations.]

### Finding seats:

In a partially occupied cinema seating plan, find a group of close seats that can host a group of friends. This problem was lifted straighted out of ACM’s ICPC annual programming contest [10], a source of inspiration that I highly recommend. [Table-valued functions, WITH ORDINALITY, LATERAL.]

### Visibility in the hills:

Perform a *maximum-scan* across the two-dimensional plane to determine object visibility in a hilly landscape. [Non-recursive CTEs, window functions.]

### Expression parsing:

Use *scans* along an input expression to check whether its subexpressions are properly parenthesized. [unnest(), WITH ORDINALITY, LATERAL, window functions.]

### Sessionization:

Given a log of system activity, try to identify sessions that can be attributed to legitimate users/intruders. [Non-recursive CTEs, window functions.]

### Run-length encoding:

Compress the pixel-based representation of an image using run-length encoding. Decompress the encoded image to restore the original. See Fig. 5a for a slide that introduces this problem scenario. [Non-recursive CTEs, window functions, string processing.]

### Landscape features:

Given a sequence of altitude measurements, identify peaks and valleys. This is a variant of a time series scenario. [Non-recursive CTEs, window functions.]

### Consecutive ranges:

Reorder a series of integers (*e.g.*, bibliographic references) to identify and compress consecutive numbers ranges. [Window functions, row numbering.]

### Linear approximation:

Partition points in a set of measurements, locally approximating sets of point values by lines. [Non-recursive CTEs, complex window functions.]

### Bulk tree traversal:

Traverse a given family of array-encoded trees, constructing an entire set of paths in one go. [Recursive CTEs, array operations.]

### Connected components:

Identify and label the connected components in an undirected graph. [Recursive CTEs, window functions.]

### Finite state machines:

Simulate the operation of a finite state machine derived from a regular expression. Parse entire batches of input strings (here: chemical formulæ) in parallel. [Recursive CTEs, window functions, string processing.]

### Sudoku solver:

Implement a brute force *generate-and-test* Sudoku solver. Derived from an example in the documentation of SQLite3 [18]. [Recursive CTEs, array processing.]

### Loose index scan:

Perform super-efficient duplicate elimination through repeated $\text{B}^{+}$tree traversals. Adapted from the *PostgreSQL* wiki [19]. [Recursive CTEs, indexes.]

### *K*-means clustering:

Identify *K* clusters in a set of points through iterated point-to-cluster assignment [11]. The core SQL query is a mere 10-liner. [Recursive CTEs, geometric operations.]

### Marching squares:

Walk the border of an unknown two-dimensional object. A classic algorithm in computer graphics and rendering [12]. Illustrates the tabular encoding of case distinction. [Recursive CTEs, LATERAL.]

### Game of Life:

Implement John Horton Conway’s classic *Game of Life* [7]. [Recursive CTEs, complex window functions.]

### Liquid flow simulation:

Simulate the flow of liquid in a tank. Based on a two-dimensional cellular automaton. Among the more complex scenarios which require the authoring of truly complex queries, typically across an entire series of slides. Fig. 5b depicts the construction of the associated recursive CTE of which the part marked  is only developed on a subsequent slide. Fig. 5c shows a rendering of various simulation time steps. [Recursive CTEs (non-linear recursion), complex window functions, LATERAL.]

### Context-free parsing:

Parse sentences of a given context-free grammar, based on the Cocke-Younger-Kasamai (or CYK) algorithm [22]. Leads to a discussion of preserving memory during recursion. [Recursive CTEs (binary recursion), string processing.]

### Spreadsheet evaluation:

Based on a tabular encoding of a spreadsheet and its formulæ, derive cell dependencies and then perform sheet evaluation. [PL/SQL, array processing, JSON processing.]

It is foreseeable that the majority of students will never be required to build parsers or simulate cellular automata with SQL. With the above computational tasks successfully implemented, however, students built confidence in both, the potential of SQL and their own ability to express these problems in a declarative, set-oriented fashion. I am positive that most students who completed the course will understand RDBMSs as capable data processors rather than mere keepers of piles of tabular data.

## Students’ Teaching Evaluation (Summer 2020)

As I write this in October 2020, the videos in the $\textit{DB2}$ and *Advanced SQL* play lists have a cumulative view count of 26 200+ (more than 3 300 hours of watch time). Viewers predominantly are from the Tübingen area, but *YouTube* analytics data and e‑mail feedback indicate that the videos have a global reach.

With the summer semester of 2020 drawing to a close in late June, the Computer Science department at University of Tübingen contacted its students to conduct the regular teaching evaluation. Survey forms were sent to the $80+$ and $90+$ students of *Advanced SQL* and $\textit{DB2}$, respectively. A sample of their responses (in German) has been reproduced below. Based on the students’ evaluation, among the 37 courses that were evaluated by the department in that semester, $\textit{DB2}$ and *Advanced SQL* ranked in the two top spots.

*Die Qualtität der Videos ist wunderbar, gerade das Unterteilen der Themen in kleinere Videosegmente ist eine sehr gute Idee. Der Mix aus Theorie (Folien) und Praxis (Shell/Queries) ist genau das was ich brauch um ein Thema gut zu durchblicken.*

*Die Veranstaltung wurde optimal digital umgesetzt. Die Videos von Herrn Grust haben eine extrem hohe Qualität.*

*Die Videos haben eine angemessene Länge und mit den großen Folien und dem kleinen Greenscreen-Grust ein sehr vorlesungsnahes Feeling - und es ist auch schön, dass sie auf YouTube im Prinzip für Alle, die es interessiert, verfügbar sind. … Wie immer wird gut erklärt und es gibt viele Beispiele, Folien mit stylischen ASCII-Grafiken (Hut ab!), …*

*Auch das Aufteilen einer Vorlesung in mehrere kleine Videos finde ich sehr gut. Im Vergleich zu anderen Vorlesungen, die ihre Inhalte immer am Stück hochladen ist es in dieser Vorlesung einfacher, den einzelnen Gedankengängen zu folgen und spezielle Themen zu wiederholen. Auch dass die Übungsblätter angeben bis zu welchem Video sie den Stoff abfragen, macht die Leistungsanforderung sehr transparent und man weiß genau welche Teile der Vorlesung man wiederholen sollte, ….*

*Dass die Vorlesung aufgezeichnet wird, statt live stattzufinden finde ich sehr erfreulich, da die Videos zu persönlich gut passenden Zeiten geschaut werden können, auch mitten in der Nacht. Außerdem kann man die Vorlesung pausieren um nachzudenken oder einer anderen Tätigkeit nachzugehen, die Wiedergabegeschwindigkeit bei Bedarf verändern und sich Inhalte wiederholt ansehen. Die Videos speziell zu dieser Veranstaltung haben außerdem eine sehr gute Länge, sodass sie sich gut einteilen lassen und kurze Erholungspausen einfach ermöglichen.*

*[Positiv war] Aufbau und Struktur der Vorlesung, Übersichtlichkeit der Videos in der Playlist ….*

*Prof. Grust erklärt in seinen Videos wirklich sehr gut und man merkt, dass er dieses Thema sehr gerne lehrt! Das motiviert natürlich nochmal ein bisschen mehr. Er ist einer der einzigen Dozenten, die sehr schnell die Umstellung auf digitale Medien (toll) hinbekommen hat,*

*On Demand Videos in Top Qualität und aufwendig erstellt. Gut erklärte Sachverhalte. Anschauliche Beispiele.*

*Ich finde die Veranstaltung sehr gut und setze mich gerne mit dem Stoff auseinander. Mir gefällt die Lehre mittels asynchroner Videos. Gerne immer so!*

*Auch wenn es wieder Präsenzlehre gibt, sollten die Videos für zukünftige Generationen auf youtube erhalten bleiben.*

*Massives Lob auch dass Übungen über Github laufen, falls Fehler/Unklarheiten in Aufgabenstellungen sind, werden Changes gepusht, das ist super! Falls dann etwas unklar wäre, [sind] die Reaktionszeiten und Antworten im Forum sehr gut.*

## Footnotes

1 *Open Broadcaster Software*, https://obsproject.com.
